# Mast Cells and Interstitial Cystitis/Bladder Pain Syndrome Revisited

**DOI:** 10.1007/s00192-025-06213-y

**Published:** 2025-07-19

**Authors:** Hannah Ruetten, Rory Ritts, Mary Namugosa, Wencheng Li, Robert Evans, Gopal Badlani, Stephen J. Walker

**Affiliations:** 1 Wake Forest Institute for Regenerative Medicine, Winston-Salem, NC 27101 USA; 2 Department of Urology, Wake Forest University School of Medicine, Winston-Salem, NC 27101 USA; 3 Department of Pathology, Wake Forest University School of Medicine, Winston-Salem, NC 27101 USA

**Keywords:** Inflammation, Pathology, TPSAB1, Urinary bladder

## Abstract

**Introduction and Hypothesis:**

There is significant variation in interstitial cystitis/bladder pain syndrome (IC/BPS) biopsy processing and reporting. The objective of this study was to review pathology reports from a large IC/BPS patient cohort to identify differences in findings. We hypothesize that variation in IC/BPS bladder biopsy reporting might be most frequent when it comes to mast-cell counts.

**Methods:**

We performed a retrospective analysis of 461 diagnostic pathology reports collected from our IRB-approved prospective study of patients diagnosed with IC/BPS at the Urology Clinic at Wake Forest Baptist Hospital from October 2011 to July 2023 (IRB00018552). Data were assigned as continuous or categorical variables. Groups were compared using Student’s *t* test, Mann–Whitney, or Chi-squared tests.

**Results:**

Staining strategy for mast-cell visualization differed between pathologists and included in order of frequency mast-cell tryptase (TPSAB1), CD117 (KIT), unspecified stain, a combination of stains, and toluidine blue. Mast-cell count was reported as a single number, range, or qualitatively. Pathologists used units of high-powered field (HPF), mm^2^, or did not specify. As expected, average mast-cell count per HPF was significantly lower than per mm^2^ across all stains (*p* < 0.0001). Average count with KIT was significantly lower than TPSAB1 (*p* < 0.0001). This trend remained significant when considering only KIT and TPSAB1 counts per HPF (*p* = 0.0007). Additionally, reports identified squamous metaplasia, acute inflammation, and/or chronic inflammation.

**Conclusions:**

There is a lack of standardization regarding histological analysis of bladder biopsies from patients with IC/BPS, leading to inconsistent data and confusion surrounding the significance of pathology report findings.

## Introduction

Interstitial cystitis/bladder pain syndrome (IC/BPS) is a condition defined by more than 6 weeks of pain arising from the bladder/pelvis that is associated with lower urinary tract symptoms [1, 2]. In 2011, Berry et al. estimated that between 3 and 8 million women in the USA experience IC/BPS symptoms based on 60-min phone interviews screening for patients that meet the case definition of IC/BPS [3]. Despite this large number of patients with an IC/BPS diagnosis and extensive studies that have been conducted, questions remain regarding the etiology and pathophysiology of IC/BPS. One pathophysiological and diagnostic aspect that has remained particularly controversial since as early as the 1980 s is the importance of mast cells in disease symptomology and progression [4–6].

Mast cells are inflammatory cells typically involved in allergic and type 1 hypersensitivity reactions [7, 8]. There are three known subtypes of mast cells in humans, named based on the presence/absence of granules containing tryptase and chymase [8]. Both mast cells containing only tryptase (MC_T_) and mast cells containing both tryptase and chymase (MC_TC_) have been found in high numbers within the bladders of patients with IC/BPS [9]. MC_T_ and MC_TC_ have unique functions. MC_T_ are T cell dependent and MC_TC_ are sensitive to compound 48/80 and substance P [8]. Further, mast cells undergo additional differentiation in their resident tissue, taking on unique organ-/tissue-specific characteristics [8].

Within the bladder, mast cells are thought to mediate an inflammatory cascade that ultimately results in pain and damage to the surrounding tissue [10]. To investigate the significance of mastocytosis in the context of IC/BPS, many researchers have attempted to quantify the number of mast cells in bladder biopsies [9, 11]. However, owing to a lack of standardization in bladder-biopsy collection and evaluation, it is exceedingly difficult to draw meaningful conclusions from the current literature.

Inclusion criteria for IC/BPS patients vary greatly from study to study. Some studies include only patients with Hunner lesions and others include all patients with IC/BPS lumped together in a single population. Assessment of mast cells in IC/BPS bladder biopsies show significant methodological inconsistency. Published studies have assessed mast cells at different layers of the bladder (lamina propria vs muscularis), with multiple types of stains (toluidine blue, mast cell tryptase [TPSAB1], CD117 [KIT]), with varying units (high powered field [HPF] vs mm^2^), and at multiple bladder locations (trigone, dome, lateral walls) [4, 5, 12, 13, 14]. These substantial variations in patient demographics and mast-cell analysis methodologies have precluded the possibility of conducting large-scale systematic reviews and meta-analyses. Because of these inconsistencies and shortfalls, we believe that revisiting the topic of mast-cell count in IC/BPS is warranted and necessary.

The objective of this study is to evaluate and summarize diagnostic pathology reports from a large cohort of patients with IC/BPS biopsied by a single surgeon with specific focus on assessing variability in mast-cell analysis. The study also seeks to explore reasons why clinicians may find these reports unclear or of limited utility. Based on these findings, we propose recommendations to standardize biopsy processing and reporting for more readily interpretable results and for more standardized data for large-scale systematic reviews and meta-analyses.

## Materials and Methods

This study was a retrospective analysis of diagnostic pathology reports obtained from a previously IRB-approved prospective study of patients with IC/BPS enrolled at the Urology Clinic at Wake Forest Baptist Hospital between October 2011 and July 2023 (IRB00018552). All study participants were included in this cross-sectional analysis. At study enrollment, patients were between the ages of 18 and 80, were diagnosed with IC/BPS in accordance with the American Urological Association guidelines [2], underwent therapeutic hydrodistension (HOD), and had a biopsy taken from the posterior bladder wall via cold-cup forceps. The clinician placed a standard pathology review request for the biopsy tissue.

The HOD and biopsy were performed while patients were under general anesthesia. HOD was performed with a uniform protocol whereby all patients had their bladders filled with sterile saline to a pressure of 100 cmH_2_O for 5 min. Data collected during HOD included anesthetic bladder capacity and the presence or absence of Hunner lesions. Additionally, demographic information such as age, body mass index (BMI), gender, and race was verified at the time of enrollment.

Patients were excluded from the study if they had a history of urethral diverticulum, bladder tuberculosis, genital herpes, genitourinary cancer, neurological conditions that could affect voiding dynamics (e.g., spinal-cord injury, stroke, Parkinson’s disease, multiple sclerosis, spina bifida), a history of cyclophosphamide treatment, radiation cystitis, or were pregnant at the time of recruitment.

To reduce bias, all pathology reports were reviewed in their original form and were not altered or reinterpreted by the research team or pathologist.

Statistical analyses were performed using GraphPad Prism (version 10.1.1). Demographic variables, clinical characteristics, and pathological data were examined for outliers and range checks were performed. Continuous variables included age, BMI, anesthetic bladder capacity, and mast-cell count. All other variables were treated as categorical data, including gender (female vs male), race (white, African American, Hispanic, and other), low bladder capacity (≤ 500 cc), Hunner lesion status, type of stain used (TPSAB1, KIT, toluidine blue, a combination of two or more stains, or unspecified), units (HPF vs mm^2^), methodology of mast-cell count (single number, a range of numbers, or qualitatively), inflammation (acute vs chronic), and the presence of squamous metaplasia. Student’s *t* tests were performed to determine associations between continuous variables. When data could not be normalized through transformation Mann–Whitney test was performed. Categorical variables were compared using Chi-squared tests. A *p* value of < 0.05 was considered statistically significant.

## Results

Data from 461 patients were included in the diagnostic pathology report review. Both low anesthetic bladder capacity [15, 16] and presence of Hunner lesions [17] are thought to be indicative of a more severe disease phenotype. Demographic information and IC/BPS disease characteristics, bladder capacity and presence of Hunner lesions can be found in Table 1.

**Table 1:** Demographic and interstitial cystitis/bladder pain syndrome (IC/BPS) classification data for 461 bladder biopsies from patients with IC/BPS

	Data
Demographic information	
Total sample population, *n (%)*	461 (100)
Gender, *n (%)*	
Female	424 (92.0)
Male	37 (8)
Race, *n (%)*	
White	396 (85.9)
African American	55 (11.9)
Hispanic	5 (0.9)
Other	5 (0.9)
BMI, average (standard deviation)	29.47 (7.68)
IC/BPS classification, *n (%)*	
Bladder capacity ≤ 500 cc	105 (22.8)
Bladder capacity > 500 cc	356 (79.2)
Hunner lesion positive	49 (10.6)
Hunner lesion negative	412 (89.4)
Bladder capacity, average (standard deviation)	812.75 (340.50

In the reports the most common description of mast-cell quantity was given as a single number (85.0%) with the units of HPF (84.6%), based on a TPSAB1 (61.0%) stain. Other stains performed included KIT (29.3%), toluidine blue (2.0%), a combination of two or more stains (3.7%), or no stain specified (4.0%). Aside from mast-cell counts, reports identified only acute inflammation (0.7%), only chronic inflammation (72.7%), and both acute and chronic inflammation (5.6%). Squamous metaplasia was found in 1.3% of samples. A more thorough summary of pathology report characteristics can be found in Table 2.

**Table 2:** Summary of diagnostic pathology report data of 461 bladder biopsies from patients with interstitial cystitis/bladder pain syndrome (IC/BPS)

	Data
Mast-cell stain, *n* (%)	
TPSAB1	281 (61.0)
KIT	135 (29.3)
Toluidine blue	9 (2.0)
Combination	17 (3.7)
Not specified	19 (4.1)
Mast-cell quantity, *n* (%)	
Single number (count)	392 (85.0)
Range	39 (8.5)
Qualitative	30 (6.5)
Field of view units, *n* (%)	
HPF	390 (84.6)
mm^2^	39 (8.5)
Unspecified	32 (6.9)
Inflammation, *n* (%)	
Only acute	3 (0.7)
Only chronic	335 (72.7)
Both acute and chronic	26 (5.6)
Squamous metaplasia	6 (1.3)
Mast-cell count, average (standard deviation)^a^	28.91 (21.20)

*TPSAB1* mast-cell tryptase, *KIT* CD117, *HPF* high-powered field

^a^Mast-cell count average is based on all values given as a single number, regardless of stain used or units of count (*n* = 392)

**Fig. 1:**
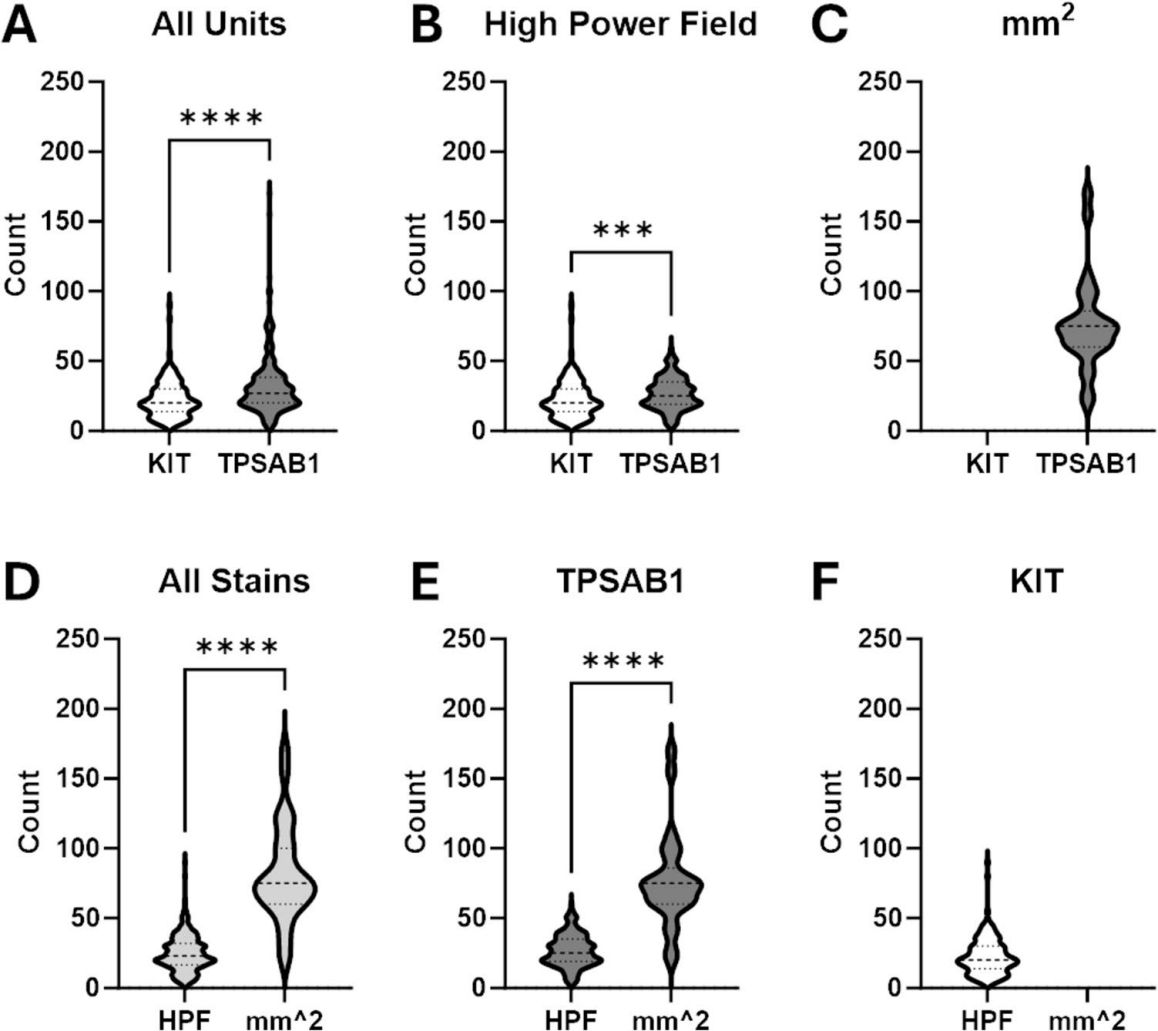
Average mast-cell count based on the type of stain used and units. We performed a retrospective review of reports from 461 patients with interstitial cystitis/bladder pain syndrome (IC/BPS) patients. A total of 392 patients had mast-cell counts given as a single value, which were used to assess the impact of stain type and units of measurement on the numerical mast-cell count number listed in the report. **A–C** A comparison of counts between biopsies stained with KIT vs TPSAB1. **D–F** A comparison of counts between those with units of HPF vs mm^2^. Pair-wise comparisons were made using a Mann–Whitney *U* test. Results are representative of *n* = 25–237. *p < 0.05 but *p* > 0.01, **p < 0.01 but > 0.001, ***p < 0.001 but > 0.0001, ****p < 0.0001

We compared mast-cell counts between the two most common stains, TPSAB1 and KIT. For these evaluations we only used counts that were reported as a single value. Using the KIT staining technique fewer mast cells were detected than with TPSAB1, both when disregarding units (22.85 ± 13.67 vs 32.03 ± 21.73; *p* < 0.0001) and when looking solely at the reports using HPF as the units (22.85 ± 13.67 vs 26.58 ± 11.47; *p* = 0.0007; Fig. 1).

Mast-cell counts reported per mm^2^ were significantly higher than those reported per HPF, both when using all stains combined (81.21 ± 34.85 vs 24.71 ± 12.67, *p* < 0.0001) and with TPSAB1 stain alone (76.16 ± 33.42 vs 26.58 ± 11.47, *p* < 0.0001; Fig. 1).

Finally, we determined if low bladder capacity (≤ 500 cc), a biomarker of bladder-centric IC/BPS, correlated with a difference in diagnostic pathology report findings. Reports from patients with low bladder capacity had a higher frequency of combined acute and chronic inflammation (17.1% vs 2.2%, *p* < 0.0001) and squamous metaplasia (3.8% vs 0.6%, *p* = 0.0099) compared with reports from patients with nonlow-capacity bladders (Table 3). Patients with low-capacity bladders were also more likely to have a clinical diagnosis of Hunner lesions (35.2% vs 3.4%, *p* < 0.0001, Table 3). There were no differences between mast-cell counts in low-bladder-capacity bladders versus nonlow-capacity bladders (Table 3).

**Table 3:** Differences in diagnostic pathology report characteristics and Hunner lesions based on low versus nonlow bladder capacity

	Bladder capacity ≤ 500 cc	Bladder capacity > 500 cc	*p* value
Acute inflammation only	1/105 (1.0%)	2/356 (0.6%)	0.6618
Chronic inflammation only	69/105 (65.7%)	266/356 (74.7%)	0.0688
Both acute and chronic	18/105 (17.1%)	8/356 (2.2%)	< 0.0001
Squamous metaplasia	4/105 (3.8%)	2/356 (0.6%)	0.0099
Hunner lesions	37/105 (35.2%)	12/356 (3.4%)	< 0.0001
KIT, average			
HPF	19.42 ± 11.57 (*n* = 19)	22.97 ± 13.96 (*n* = 115)	0.3133
mm^2^	–	–	–
TPSAB1, average			
HPF	25.39 ± 12.20 (*n* = 59)	25.09 ± 11.58 (*n* = 172)	0.9780
mm^2^	82.40 ± 39.54 (*n* = 10)	71.94 ± 32.64 (*n* = 18)	0.4579

*KIT* CD117, *TPSAB1* mast-cell tryptase, *HPF* high-powered field

Patients with IC are also subclassified by the presence of Hunner lesions. Reports from patients with Hunner lesions had a higher frequency of combined acute and chronic inflammation (24.5% vs 3.4%, *p* < 0.0001), and squamous metaplasia (6.1% vs 0.7%, *p* = 0.0016) compared with reports from patients without Hunner lesions (Table 4). There was a trend toward increased numbers of TPSAB1-positive mast cells in Hunner lesion-positive versus -negative biopsies when cell counts were performed per HPF (30.16 ± 11.19 vs 26.08 ± 11.45, *p* = 0.0828) but this did not hold true when TPSAB1-positive mast cells were counted per mm^2^ (97.33 ± 4.62 vs 73.27 ± 34.67, *p* = 0.2504) (Table 4). There were no differences in KIT-positive mast-cell counts between Hunner lesion-positive or -negative patients (Table 4).

**Table 4:** Differences in diagnostic pathology report characteristics based on Hunner lesion status

	Hunner lesion positive	Hunner lesion negative	*p* value
Acute inflammation only	1/49 (2.0%)	2/412 (0.5%)	0.2005
Chronic inflammation only	32/49 (65.3%)	303/412 (73.5%)	0.2213
Both acute and chronic	12/49 (24.5%)	14/412 (3.4%)	< 0.0001
Squamous metaplasia	3/49 (6.1%)	3/412 (0.7%)	0.0016
Bladder capacity	389.0 ± 184.6	849.7 ± 331.9	< 0.0001
KIT, average			
HPF	19.25 ± 12.83 (*n* = 8)	23.08 ± 13.74 (*n* = 122)	0.4016
mm^2^	–	–	–
TPSAB1, average			
HPF	30.16 ± 11.19 (*n* = 25)	26.08 ± 11.45 (*n* = 177)	0.0828
mm^2^	97.33 ± 4.62 (*n* = 3)	73.27 ± 34.67 (*n* = 22)	0.2504

*KIT* CD117, *HPF* high-powered field, *TPSAB1* mast-cell tryptase

## Discussion

Our study highlights significant inconsistencies in mast-cell-staining techniques in bladder biopsies from patients with IC/BPS. Among 461 biopsies stained for mast cells, pathologists used TPSAB1 in ~ 60% of cases, KIT in 30% of cases, and either toluidine blue, a combination of two or more stains, or did not specify in the remaining 10% of cases. Toluidine blue is an inexpensive and quick stain to perform for mast cells. It relies on the mast cells having a metachromatic reaction to the dye, causing them to appear purple to red [18]. For the metachromatic reaction to appear in stark contrast to the background light-blue staining, a dilute solution of the dye is used. The pH of the dye is also important, with normal mast cells staining normally at pH < 3.5; however, malignant mast cells do not stain at this lower pH [18]. Further, individual granules are stained with this technique, allowing for assessment of ongoing degranulation by observing granules in the extracellular space immediately surrounding a cell with few granules. The only potential downside is that toluidine blue does not distinguish between mast cells and basophils, although this is generally not a concern when looking at tissue biopsies. In contrast, mast cells express KIT and basophils do not; however, mast cells are not the only cell type that expresses KIT. KIT immunohistochemical staining also labels germ cells, melanocytes, stem cells, and many other epithelial and stromal cell types. Therefore, KIT is often used in combination with protein tyrosine phosphatase receptor type C to ensure that labeled cells are of hematopoietic origin. Further, a portion of mast cells specific to many tissue types, including the bladder, are KIT negative [19]. When mast cells are immunopositive for KIT the staining is cytoplasmic. Tryptase alpha-1 and tryptase beta-1 (TPSAB1; also known as mast-cell tryptase) is one of two major proteases found in mast-cell granules. Immunohistochemistry for TPSAB1 should identify both mast-cell subtypes identified in human bladder [9]. We found that staining with KIT was associated with significantly lower numbers of mast cells than staining with TPSAB1. This is consistent with the known fact that a subset of bladder mast cells are KIT immunonegative [19].

Mast-cell count was only described quantitatively as a single number in 85% of biopsies. The remainder either used a range of numbers (e.g., 12–20) or used qualitative expressions such as “low” or “high.” Although all seemingly valid pathology assessments, they complicate interpretation of the report by clinicians who are less familiar with the pathology lexicon and what that really means. Pathologists often use ranges when there is a high variability in the density of what is being counted between regions on the sample. For example, there is an aggregate of mast cells around a single vessel and in this location the count is high, but in another area of the sample there are no mast cells at all. Other pathologists may use a range if they are just quickly glancing through several fields to get a general idea but not actually writing down a count and calculating an exact value. A clinician likely more easily interprets single numbers, even though they may not be as important as a granular value. As long as the assessment is being performed in the same way, a clinician can notice trends and associations between high or low counts and patient characteristics.

The units for mast-cell counts were also inconsistent among reports, with the majority using either HPF (84.6%) or mm^2^ (8.5%). It is likely logical for most pathologists who know the approximate size in mm^2^ of each field of view on their scope that counts given in mm^2^ would be higher than those per HPF. However, this may not be readily apparent to clinicians who are just reading a number on a report. Most ×400 fields are around 0.2–0.24 mm^2^ but the exact dimensions vary by the scope eyepiece and specific objective. When viewing reports, this difference appears to account for the greatest variability seen in mast-cell counts. For example, when looking at biopsies stained only with TPSAB1, a mm^2^ field of view resulted in 76 cells on average compared with 27 cells with HPF, which is a three-fold statistically significant difference. If clinicians are not catching the change in units it is going to greatly impact their interpretation of the mast-cell count. Because mm^2^ is a fixed unit independent of microscope variation, it provides the most standardized reporting method for consistent and comparable results.

Unfortunately, the discrepancies in mast-cell assessment methodology are not limited to our institution and instead reflect broad findings in the published literature. Possibly the best example of this is outlined in an in-depth review by Theoharides et al., which summarizes dozens of studies that employ various mast-cell stains, fields of view, and tissue locations to assess mast-cell count [11]. As there has been so much variation in how researchers and pathologists report mast-cell count, it is difficult for the community to understand their significance. In combination with the literature, our study demonstrates the need for standardization regarding the specific stain, units, and bladder/tissue locations to assess mast-cell count in these patients. We recommend using a TPSAB1 (also known as mast-cell tryptase) immunostain to identify mast cells in bladder tissue. We recommend that pathologists report mast-cell counts as a single number per mm^2^. If specific locations are assessed that may provide additional information, this information should be included, but we recommend that an overall average for the entire tissue is given for broad comparability. A summary of our recommendations for standardizing mast-cell counts in biopsies from patients with IC/BPS can be found in Table 5.

**Table 5:** Recommendations for assessing and reporting mast cell counts from IC/BPS bladder biopsies

Assessing Mast Cells in IC/BPS patient bladder biopsies		
Stain	TPSAB1 (aka mast-cell tryptase)	Use an immunohistochemical stain for TPSAB1 to identify mast cells in tissue sections. KIT does not stain all bladder mast cells
Units	Mast cells per mm^2^	Because “high-power field” varies from scope to scope report counts per mm^2^. Steps to determine field of view in mm^2^: take your eyepiece field number (e.g., 20 mm) and divide it by the objective magnification (e.g., ×40 objective = ×40). This will give you the field-of-view diameter (20 mm/40 = 0.5 mm). Use the field-of-view diameter to calculate area using the formula for the area of a circle (πr^2^; e.g., above 0.20 mm^2^). take your counted mast-cell number per high-power field and divide by the field-of-view area
Count	Single number of average mast-cell density for entire tissue present	Give at minimum a single number noting average density of mast cells in the entire sample. As long as this is given, additional information, including counts for each region, is valuable

Our study also assessed differences in pathology report findings in patients with a low bladder capacity and those with Hunner lesions. Overall, a finding of combined acute and chronic inflammation and squamous metaplasia was significantly more common in patients with a low bladder capacity and in patients with Hunner lesions. In addition, there was some overlap between patients with Hunner lesions and patients with a low bladder capacity that resulted in similar findings for both populations. In general, a diagnosis of acute inflammation indicates neutrophilic and/or granulocytic inflammation and a diagnosis of chronic inflammation indicated lymphoplasmacytic inflammation. In the future a more specific look at individual immune cell types (neutrophils, lymphocytes, plasma cells, and macrophages) within specific bladder layers (urothelium, submucosa, detrusor) would provide more information on the role of inflammation in disease subtypes.

Past studies have found that bladders from patients with Hunner lesions have greater bladder mast-cell infiltration than bladders from those without Hunner lesions and non-inflamed control bladder tissues [20–22], whereas other studies found the reverse results [23]. Another study found that mast-cell infiltration in bladder tissue from patients with Hunner lesions was comparable with that in the bladder tissue of patients with other chronic cystitis conditions with similar levels of inflammation [24], suggesting that mast-cell density simply mirrors the degree of inflammation in the bladder. We found that a statistically increased mast-cell count was not associated with a specific IC/BPS subtype. However, there was a trend (*p* = 0.0828) toward increased mast cells in Hunner lesion-positive biopsies with samples stained with TPSAB1 and counted per HPF, which would be consistent with samples with more inflammation having greater mast-cell infiltration. This trend did not hold true for TPSAB1 counts based on mm^2^ units but that was likely due to the very small *n* value for the Hunner-lesion population with this assessment (*n* = 3), which underpowered the statistical analysis. This is an interesting finding in need of further evaluation.

Compared with a previous study with lower *n* values (*n* = 14 IC/BPS, *n* = 4 controls), we found similar average mast-cell counts using TPSAB1 per mm^2^ to their general IC/PBS group using the same stain and units [9]. This supports a role for mast cells in the syndrome in general rather than mast cells being associated with a specific disease etiology or a specific subset of patients. Further work is needed to evaluate the subtypes and activation profiles of mast cells in IC/BPS, which may have a more specific role in disease etiology and progression.

A limitation and a strength of this study is that it was a retrospective study of diagnostic pathology reports. Because we assessed diagnostic pathology reports, we unfortunately cannot give clear expectations for expected mast-cell counts based on a single method of assessment in this large patient population. Biopsies were reviewed and reports were written for diagnostic purposes, meaning that reports were written by many different pathologists and we had no control over how they assessed the slide or wrote their report. However, despite this limitation, we have compiled valuable data that are representative of what a single clinician receives back from the biopsies he/she collects.

Overall, we identified that a combination of acute and chronic inflammation as well as squamous metaplasia is significantly more common in patients with a low bladder capacity and patients with Hunner lesions. Further work is needed to evaluate the specific inflammatory cells and immune signatures of these IC/BPS subtypes, in addition to the activation profiles of mast cells, which may have more specific roles in disease etiology and progression. We also highlight a lack of standardization regarding diagnostic histological analysis of bladder biopsies from patients with IC/BPS and recommend that in the future pathologists use a mast-cell count per mm^2^ based on a TPSAB1 stain.

## Data Availability

The data that support the findings of this study are available on request from the corresponding author. The data are not publicly available due to privacy or ethical restrictions.
